# Altered immune pathways in patients of temporal lobe epilepsy with and without hippocampal sclerosis

**DOI:** 10.1038/s41598-024-63541-7

**Published:** 2024-06-13

**Authors:** Xiang-Qian Che, Shi-Kun Zhan, Jiao-Jiao Song, Yu-Lei Deng, Wei-Liu, Peng-Huang, Jing-Zhang, Zhan-Fang Sun, Zai-Qian Che, Jun Liu

**Affiliations:** 1 Department of Neurology & Neuroscience Institute, Ruijin Hospital affiliated to Shanghai Jiao Tong University School of Medicine, Shanghai, China; 2 Department of Neurosurgery, Centre for Functional Neurosurgery, Ruijin Hospital, Shanghai Jiao Tong University School of Medicine, Shanghai, China; 3 Department of Teaching Office, Shanghai Children’s Hospital, School of Medicine, Shanghai Jiao Tong University, Shanghai, China; 4 Department of Neurology, Shandong Provincial Hospital Affiliated to Shandong First Medical University, Jinan, China; 5 Department of Emergency, Ruijin Hospital, School of Medicine, Shanghai Jiaotong University, Shanghai, China

**Keywords:** Immune pathway, RNA, Temporal lobe epilepsy, Hippocampal sclerosis, Neuroscience, Neurology

## Abstract

Over the past decades, the immune responses have been suspected of participating in the mechanisms for epilepsy. To assess the immune related pathway in temporal lobe epilepsy (TLE), we explored the altered immune pathways in TLE patients with and without hippocampal sclerosis (HS). We analyzed RNA-seq data from 3 TLE-HS and 3 TLE-nonHS patients, including identification of differentially expressed RNA, function pathway enrichment, the protein–protein interaction network and construction of ceRNA regulatory network. We illustrated the immune related landscape of molecules and pathways on human TLE-HS. Also, we identified several differential immune related genes like HSP90AA1 and SOD1 in TLE-HS patients. Further ceRNA regulatory network analysis found SOX2-OT connected to miR-671-5p and upregulated the target gene SPP1 in TLE-HS patients. Also, we identified both SOX2-OT and SPP1 were significantly upregulated in five different databases including TLE-HS patients and animal models. Our findings established the first immune related genes and possible regulatory pathways in TLE-HS patients and animal models, which provided a novel insight into disease pathogenesis in both patients and animal models. The immune related SOX2-OT/miR-671-5p/SPP1 axis may be the potential therapeutic target for TLE-HS.

## Introduction

Epilepsy is a neurological disorder characterized by recurrent seizures resulting from abnormal excessive or synchronous neuronal activity in the brain^1^. Recent research has highlighted the intricate relationship between the immune system and epileptogenesis, as well as the effects of antiepileptic therapies. RNA-Seq studies have demonstrated significant enrichment of astrocytic and microglial genes, along with downregulation of neuron specific genes in the hippocampus of patients with temporal lobe epilepsy (TLE)^2^. Furthermore, activated glial cells have been found to produce inflammatory cytokines after seizure induction in both animal models and human chronic epileptic tissues^3–5^. Notably, immune-mediated neuronal damage has been identified as a key factor in the development of seizure activity in lupus encephalopathy^6^. Several case studies have already demonstrated the efficacy of anti-inflammatory treatments in epilepsy management^7–10^. For instance, an open-label study involving thirty-seven children with intractable epilepsy investigated the effectiveness of intravenous immunoglobulin (IVIg) treatment. After 15 months of IVIg therapy, 43% of the patients experienced a reduction of more than 50% in seizure frequency, and 15% became seizure-free^11^. These findings have led to the growing recognition of the immune pathways in the mechanisms participating in epilepsy over the past few decades.

TLE is a form of epilepsy characterized by recurrent seizures originating from the temporal lobe, accounting for approximately one-third of all epilepsy cases. Hippocampal sclerosis (HS) is the most common histopathological finding in TLE patients^12^. In a large study evaluating the types of epilepsy in patients who underwent epilepsy surgery, HS was the primary pathological diagnosis, accounting for 36.4% of all cases^13^. Recent clinical observations and experimental evidence have shed light on the evidence of both the innate and adaptive immune response activation in HS brain tissues^14,15^. The innate immune response in TLE-HS involves upregulation of astrocytes, neurons, microglia, interleukin-1a, interleukin-1b, and other inflammatory proteins^3,16,17^. The adaptive immune response in TLE-HS involves the presence of CD3-positive, CD4-positive and CD8-positive T-cells^18^. However, the precise molecular mechanisms and neuronal immune networks in TLE-HS are relatively less studied.

Despite the importance of immune-related pathways in TLE with and without HS, there are few studies directly focusing on this topic, making it a challenging area in human epileptic research. In our present study, we sought to explore immune related genes and competitive endogenous RNA (ceRNA) network in HS. Therefore, we conducted extensive bioinformatic analysis on RNA-seq data obtained from three TLE-HS and three TLE-nonHS patients.

## Materials and methods

### Clinical information and tissue samples

Patients had intractable complex partial seizures by the occurrence of a minimum of two seizures every month despite therapy with two AEDs at maximum tolerated doses. Human hippocampus samples were obtained from patients with medically intractable TLE who underwent resection of presumed epileptogenic zone at our neurosurgical center following clinical seizure monitoring and systematic electrophysiological as well as imaging assessment. Two neuropathologists independently reviewed each hippocampal specimen to examine whether HS and neuronal loss were present or not. Briefly, the excised hippocampal specimen was placed above nitrogen vapor for programmed cooling and eventually frozen in liquid nitrogen. A total of 5 TLE-HS (ILAE-1) and 5 TLE-nonHS samples were collected. We randomly chose 3 TLE-HS and 3 TLE-nonHS samples for RNA-Seq. The patients’ age at surgery ranged from 18 to 47 years old (mean 28.2 ± 10.6 years old). Average duration of epilepsy before surgery was ranged from 4 to 27 years (mean 13.2 ± 7.7 years). This study received prior approval by the institutional review board and the ethics committee of the Ruijin Hospital, Shanghai Jiao Tong University. All patients have approved the use of their tissue for scientific purposes particularly in this study by signing informed consent.

### Difference analysis of RNAs in TLE

Total RNA was extracted from frozen hippocampus tissues using miRNeasy Micro Kit (Cat#217084, Qiagen), and RNA quality was checked using an Agilent Bioanalyzer 4200 (Agilent technologies, Santa Clara, CA, US). Firstly, ribosomal RNA (rRNA) was removed by magnetic bead. Subsequently, sequencing libraries were generated using VAHTS^®^ Universal V6 RNA-seq Library Prep Kit for Illumina (NR604, Vazyme, Nanjing, China) according to the manufacturer’s instruction. The sequencing was performed on Illumina Nova seq platform (Illumina, San Diego, CA, USA). For small RNA libraries, sequencing libraries were constructed by TruSeq Small RNA Sample Prep Kits for Illumina (Cat #RS-200-0012) according to manufacturer’s instruction. Then sequencing was performed on an Illumina NovaSeq platform (Illumina, San Diego, CA, USA). Sequence data that support the findings of this study have been uploaded to the GEO, and the number is GSE255223.

Raw data was processed through Seqtk. In this step, the raw data was cleaned by removing reads containing adaptors, contaminants, and low-quality reads. Additionally, the Q30 and GC content were calculated to estimate the quality of clean reads. Differential expression analysis from RNA-seq (lncRNA, miRNA and mRNA) data was performed using Limma in the R package, and significant differential expression RNAs were determined when the results showed | log 2FoldChange|> 1 and P value < 0.05. Volcano plots and heatmap of results were applied for differential analysis.

### Functional enrichment analysis of differential genes

The potential functional enrichment of mRNA was explored using DAVID (https://david-d.ncifcrf.gov/) for Gene Ontology (GO) and Kyoto Encyclopedia of Genes and Genomes (KEGG) analysis^19^. The top20 pathways were selected based on p-value < 0.05 and count > 2, which considered as significant enrichment functions.

### Construction of protein–protein interaction (PPI) network

We used the differential mRNA to construct a PPI network through Retrieval of Interacting Genes/Proteins (STRING) database (Version11.5; http://www.string-db.org/), and the cluster genes of the PPI network were calculated using Cytoscape software (version 3.6.1; https://cytoscape.org).

### Analysis of immune related genes in epilepsy samples

Differential expression genes (DEGs) of each cluster were screened to identify immune related genes (IRGs) based on the ImmPort database (https://www.immport.org/home), and IRGs within the DEGs were selected for further research. Expression of different lncRNAs and mRNAs was analysed using Pearson’s correlation coefficient. A p-value < 0.05 was considered statistically significant. Predicted target genes were assigned based on KEGG and then constructed a PPI network through STRING database.

### Establishment of the ceRNAs network

Prediction of microRNA targets was conducted using miRWalk 3.0 (http://mirwalk.umm.uni-heidelberg.de/) for miRNA-mRNA interaction, and lncbase2.0 (http://carolina.imis.athena-innovation.gr/diana_tools/web/index.php?r=lncbasev2%2Findex-predicted) for miRNA-lncRNA interaction. According to ceRNA hypothesis, the downregulated ceRNA sub-network consisted of down-regulated differential expression lncRNAs (DElncRNAs), upregulated differential expression miRNAs (DEmiRNAs) and down-regulated differential expression mRNAs (DEmRNAs). Whereas upregulated ceRNA sub-network was comprised of up-regulated DElncRNAs, down-regulated DEmiRNAs, and up-regulated DEmRNAs. The ceRNA network was visualized with Cytoscape software^20^.

### Quantitative reverse transcription-polymerase chain reaction analysis

To confirm the lncRNA-mRNA pathway, real-time reverse transcription-polymerase chain reaction (RT-PCR) was used to detect lncRNA and mRNA expression, which was performed on an ABI, Steponeplus Multicolor Real-Time PCR Detection System. GAPDH was used as an endogenous control for lncRNA and mRNA detection. The RT-PCR cycle was 98 ℃ for 2 min, followed by 40 cycles of 95 ℃ for 15 s and 60 ℃ for 30 s, and a final melting curve analysis (60–95 ℃) was included. RT-PCR results were quantified using the 2ΔΔct method against GAPDH for normalization. Data represent means from three experiments.

### Gene expression database acquisition and SOX2-OT/miR-671-5p/SPP1 *axis* analysis

For TLE-HS patients, two data files were downloaded from the National Center for Biotechnology Information (NCBI) Gene Expression Omnibus(GEO) public database (https://www.ncbi.nlm.nih.gov/geo/), including GSE 71,058 (5 TLE-HS patients and 7 TLE-nonHS patients. GPL11154 as a Series Matrix File, Illumina HiSeq 2000) and GSE205661 (6 TLE-HS patients and 9 normal temporal or parietal cortices from traumatic brain injury patients. GPL13534/ GPL18402/ GPL19072, Illumina HumanMethylation450 BeadChip/ Unrestricted_Human_miRNA_V19.0_Microarray/ Agilent-052909 CBC_lncRNAmRNA_V3).

For the rat model of TLE-HS, one data file was downloaded from the GEO public database: GSE1834 (1, 6, 24, 72 and 240 h after kainate induced seizures at P30 compared to P15. GPL85, Affymetrix Rat Genome U34 Array).

For the mouse model of TLE-HS, two data files were downloaded from the GEO public database, including GSE73878 (kainate or saline was injected unilaterally into the dorsal hippocampus of 12 week old C57BL/6 J mice, and the ipsilateral and contralateral hippocampi were isolated 7, 28 and 60 d later. GPL6885, Illumina MouseRef-8 v2.0 expression beadchip) and GSE88992 (intrahippocampal microinjection of kainate was used in parallel with saline-injected animals as controls. The animals were decapitated 6, 12 or 24 h post injection. GPL1261, Affymetrix Mouse Genome 430 2.0 Array).

We calculated the differential genes between the disease group and control using the edgeR algorithm in the R package for RNA-seq sequencing data, and using the Limma algorithm in the R package for chip data. We observed the differential expression of SOX2-OT, miR-671-5p and SPP1, while also selected | log 2FoldChange|> 1 and P value < 0.05 as the differential expression analysis.

### Ethics statement

We confirm that we have read the journal’s position on issues involved in ethical publication and affirm that all methods were carried out in accordance with Declaration of Helsinki.

## Results

### Identification of differentially expressed mRNA, lncRNA and miRNA

**Figure 1:**
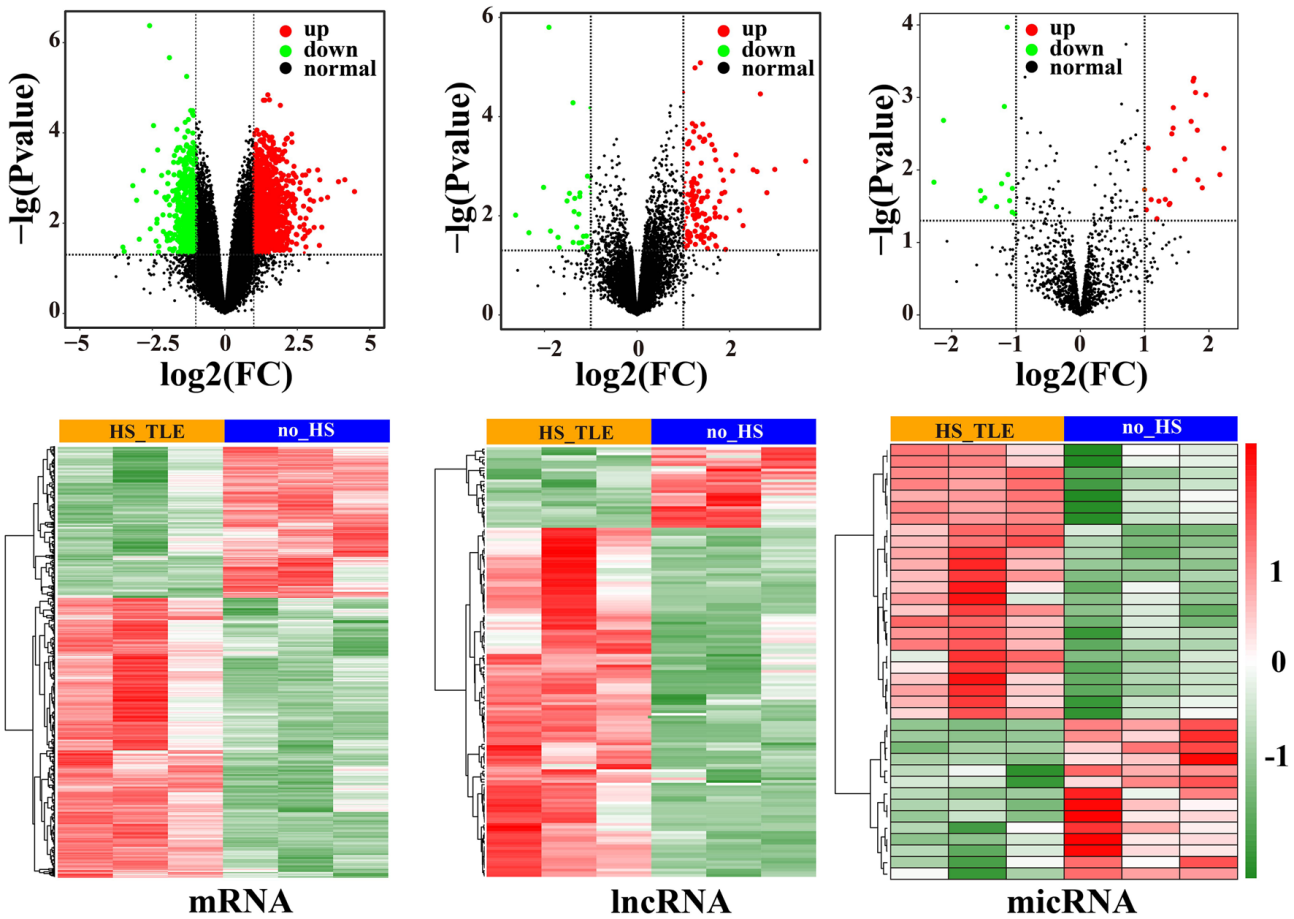
The identification of differentially expressed RNA in hippocampal sclerosis. The volcano plot of DEmRNAs, DElncRNAs, and DEmiRNAs and heatmap of DEmRNAs, DElncRNAs, and DEmiRNAs. *DE* differential expression.

To identify DEmRNAs, DElncRNAs and DEmiRNAs between TLE-HS and TLE- nonHS, we performed differential expression analysis and identified 1726 DEmRNAs (1121 up-regulated and 605 down-regulated), 160 DElncRNAs (130 up-regulated and 30 down-regulated), and 38 DEmiRNAs (24 up-regulated and 14 down-regulated) across the transcriptomes of these two groups based on the criteria of | log2FoldChange|> 1 and p-value < 0.05. The volcano plot was utilized to visualize differential expressed genes and heatmap analysis showed significance difference levels in global gene expression between TLE-HS vs TLE-nonHS groups (Fig. 1).

### Function and pathway enrichment analysis

**Figure 2:**
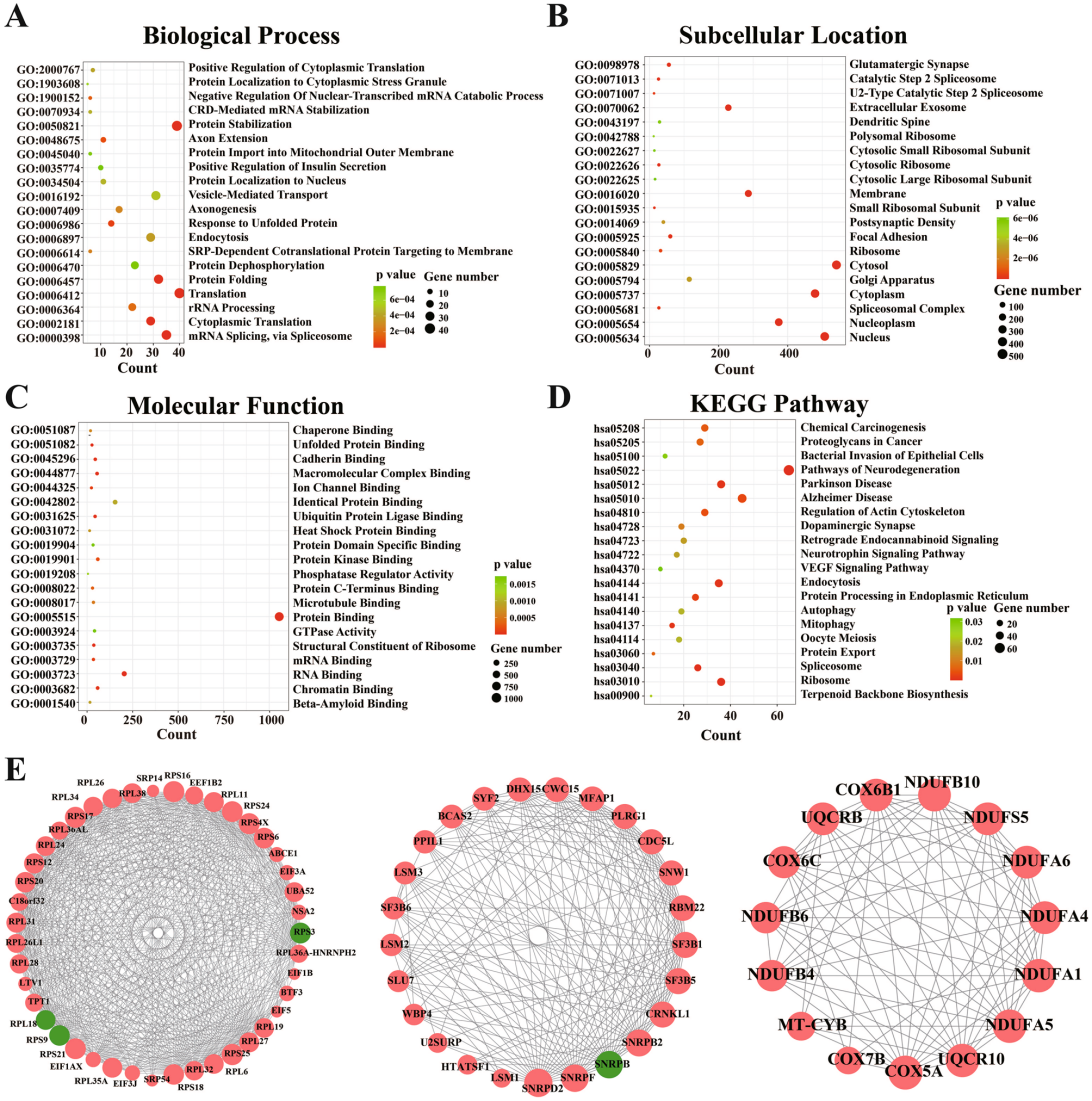
Gene ontology enrichment and KEGG pathway analysis of DEGs in hippocampal sclerosis. (**A**) biological processes, (**B**) subcellular location, (**C**) molecular function, (**D**) KEGG pathways. The size of circle represents gene number. Different colour of circles represents different p value. (**E**) The sub-PPI networks identification in hippocampal sclerosis. Cluster 1 and cluster 2 are related to information processing, cluster 3 is related to oxidative phosphorylation and neurodegenerative disease. *DEGs* differential expression genes.

The biological roles of these genes were evaluated using GO and KEGG pathway analysis. The enrichment analyses displayed DEGs significantly enriched in 143 GO biological processes (BP) (Fig. 2A), 111 GO subcellular location (Fig. 2B), 71 GO molecular function (MF) (Fig. 2C) and 25 KEGG pathways (Fig. 2D). The DEGs were significantly enriched in pathways related to information processing and cellular processes including “Ribosome”, “Spliceosome”, “Autophagy”, “VEGF signaling pathway”, “Focal adhesion”; neurodegenerative disease including “Pathways of neurodegeneration”, “Parkinson disease”, “Alzheimer disease”, “Neurotrophin signaling pathway”; and immune system including “Toll-like receptor signaling pathway”, “NOD-like receptor signaling pathway”, “RIG-I-like receptor signaling pathway”, “C-type lectin receptor signaling pathway”, “JAK-STAT signaling pathway”, “Hematopoietic cell lineage”, “Natural killer cell mediated cytotoxicity”, “IL-17 signaling pathway”, “Th1 and Th2 cell differentiation”, “Th17 cell differentiation”, “T cell receptor signaling pathway”, “B cell receptor signaling pathway”, “Fc epsilon RI signaling pathway”.

### The PPI network of the DEGs

Using the PPI score was 4 as parameters, a PPI network was established, containing 608 nodes and 2221 interactions. Top ten nodes with high degrees were UBA52 (degree = 72), RPS16 (degree = 44), RPS3 (degree = 44), RPS9 (degree = 44), RPS6 (degree = 44), HRAS (degree = 44), RPS24 (degree = 42), RPS18 (degree = 42), UBB (degree = 40), RPL11 (degree = 40). To obtain data of high quality, three sub-PPI networks were grabbed by ClusterONE (p < 1.0E-4). Further, 3 PPI clusters identified were shown in Fig. 2E. Cluster 1 and cluster 2 are related to information processing, cluster 3 is related to oxidative phosphorylation and neurodegenerative disease (Table 1).

**Table 1:** The PPI clusters in hippocampal sclerosis.

Cluster	Term	Count	P value
Cluster1	hsa03010: Ribosome	29	3.81E–47
Cluster1	hsa05171: Coronavirus disease—COVID-19	29	4.09E–42
Cluster2	hsa03040: Spliceosome	21	7.15E–35
Cluster3	hsa00190: Oxidative phosphorylation	15	5.23E–26
Cluster3	hsa05022: Pathways of neurodegeneration—multiple diseases	15	4.43E–18
Cluster3	hsa01100: Metabolic pathways	15	6.91E–11
Cluster3	hsa05012: Parkinson disease	15	1.10E–21
Cluster3	hsa05010: Alzheimer disease	15	2.09E–19

### Enrichment function analysis of immune related differential expression genes

**Figure 3:**
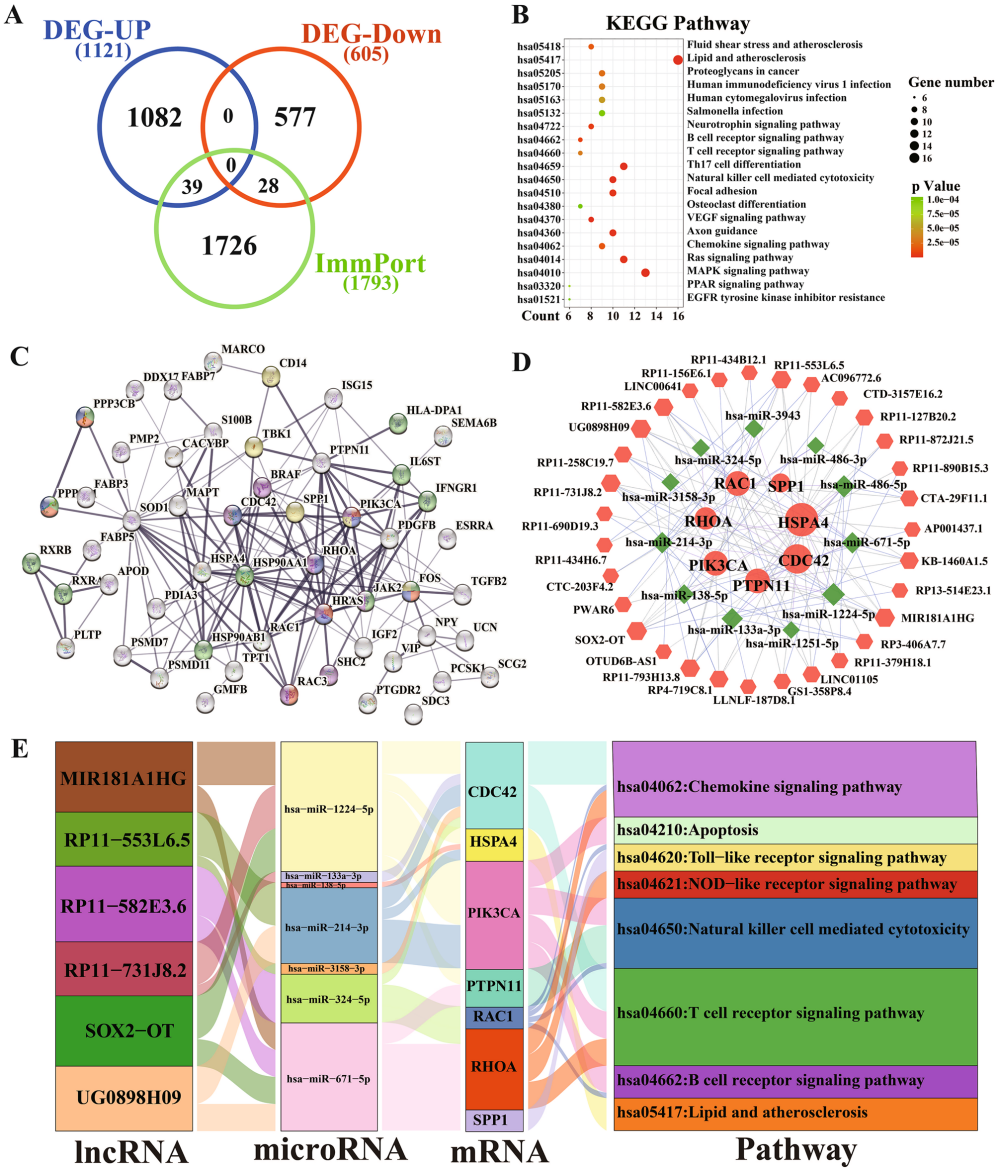
The identification and network analysis of IRGs in hippocampal sclerosis. (**A**) Veen diagram of compound targets of immune-related genes and DRGs (39 up-regulated and 28 down-regulated). (**B**) KEGG pathway analysis of IRGs. The size of circle represents gene number. Different colour of circles represents different adjusted p value. (**C**) PPI network of the overlapped IRGs. Green nodes, hsa04659: Th17 cell differentiation pathway; red nodes, hsa04662: B cell receptor signaling pathway; blue nodes, hsa04660: T cell receptor signaling pathway; yellow nodes, hsa04620: Toll-like receptor signaling pathway; purple nodes, hsa04062: Chemokine signaling pathway. (**D**) The immune-related lncRNA-miRNA-mRNA ceRNA network in hippocampal sclerosis. (**E**) Sankey diagram of the key six lncRNA regulatory mechanism. Hexagon represent DElncRNAs, rhombus represent DEmiRNAs, and circles represent DEmRNAs. *DRGs* differential expression genes, *IRGs* immune related genes, *DE* differential expression.

There were 1793 immune-related genes (IRGs) in the ImmPort database, and 67 differential DRGs (39 up-regulated and 28 down-regulated) were obtained via intersection of DEGs with ImmPort database (Fig. 3A). The differentially expressed 67 IRGs were significantly enriched in 78 pathways (Fig. 3B) and top 20 were shown in Table 2. The IRGs pathway analysis showed that they are parts of immune system including “Th17 cell differentiation”, “Natural killer cell mediated cytotoxicity”, “B cell receptor signaling pathway”, “Chemokine signaling pathway”, “T cell receptor signaling pathway”, and signal transduction including “VEGF signaling pathway”, “MAPK signaling pathway”, “Ras signaling pathway”, “Focal adhesion”. From these findings, we can deduce that immune response and signal transduction are important in the pathogenesis of TLE-HS.

**Table 2:** The KEGG pathways of IRGs in hippocampal sclerosis (TOP20).

Term	Count	P value
hsa05417: Lipid and atherosclerosis	16	8.92E–13
hsa04659: Th17 cell differentiation	11	5.75E–10
hsa04650: Natural killer cell mediated cytotoxicity	10	4.55E–08
hsa04370: VEGF signaling pathway	8	4.84E–08
hsa04010: MAPK signaling pathway	13	1.06E–07
hsa04014: Ras signaling pathway	11	1.03E–06
hsa04360: Axon guidance	10	1.06E–06
hsa04510: Focal adhesion	10	2.43E–06
hsa04722: Neurotrophin signaling pathway	8	6.06E–06
hsa04662: B cell receptor signaling pathway	7	8.45E–06
hsa04062: Chemokine signaling pathway	9	1.60E–05
hsa05418: Fluid shear stress and atherosclerosis	8	1.69E–05
hsa05205: Proteoglycans in cancer	9	2.57E–05
hsa05170: Human immunodeficiency virus 1 infection	9	3.27E–05
hsa04660: T cell receptor signaling pathway	7	3.32E–05
hsa05163: Human cytomegalovirus infection	9	5.00E–05
hsa03320: PPAR signaling pathway	6	7.96E–05
hsa01521: EGFR tyrosine kinase inhibitor resistance	6	1.02E–04
hsa05132: Salmonella infection	9	1.02E–04
hsa04380: Osteoclast differentiation	7	1.06E–04

*IRGs* immune related genes.

### PPI network construction of IRGs

To explore the relation, we used the STRING 11.5 database to construct a PPI network of IRGs, containing 54 nodes and 170 interactions (Fig. 3C). The results showed that the top 15 interacted genes included HSP90AA1 (degree = 21), SOD1 (degree = 19), HRAS (degree = 17), JAK2 (degree = 16), HSPA4 (degree = 16), FOS (degree = 16), RHOA (degree = 14), PIK3CA (degree = 13), PTPN11 (degree = 13), HSP90AB1 (degree = 12), CDC42 (degree = 12), PDGFB (degree = 11), RAC1 (degree = 10), SPP1 (degree = 8), IFNGR1 (degree = 8), among which HSP90AA1 and SOD1 are the most interactive IRGs. These results further confirmed that IRGs including HSP90AA1 and SOD1 played important roles in the pathological process of TLE-HS.

### Construction of lncRNA-miRNA-mRNA ceRNA regulatory network

These top 15 interacted genes with high degrees were used for further ceRNA regulatory network analysis. We firstly predicted DEmiRNAs-DEmRNAs through miRWalk database, and detected 75 predicted DEmiRNAs-DEmRNAs pairs including 22 predicted DEmiRNAs. Secondly, we detected a total of 185 lncRNA-miRNA pairs via lncBase v2 contained 66 DElncRNAs for further analysis. Ultimately, based on the integration of 75 DEmiRNA-DEmRNA pairs, 66 DElncRNAs, 22 DEmiRNAs, and 15 DEmRNAs were incorporated into the ceRNA regulatory network. According to ceRNA theory, the ceRNA network composed of 49 nodes (31 over-expressed DElncRNAs, 11 underexpressed DEmiRNAs, and 7 over-expressed DEmRNAs), and was visualized in Fig. 3D. Then the top six DElncRNAs constructed a Sankey diagram based on the lncRNA-miRNA-mRNA pathway relationship, as shown in Fig. 3E.

### Validation of key differentially expressed genes

**Figure 4:**
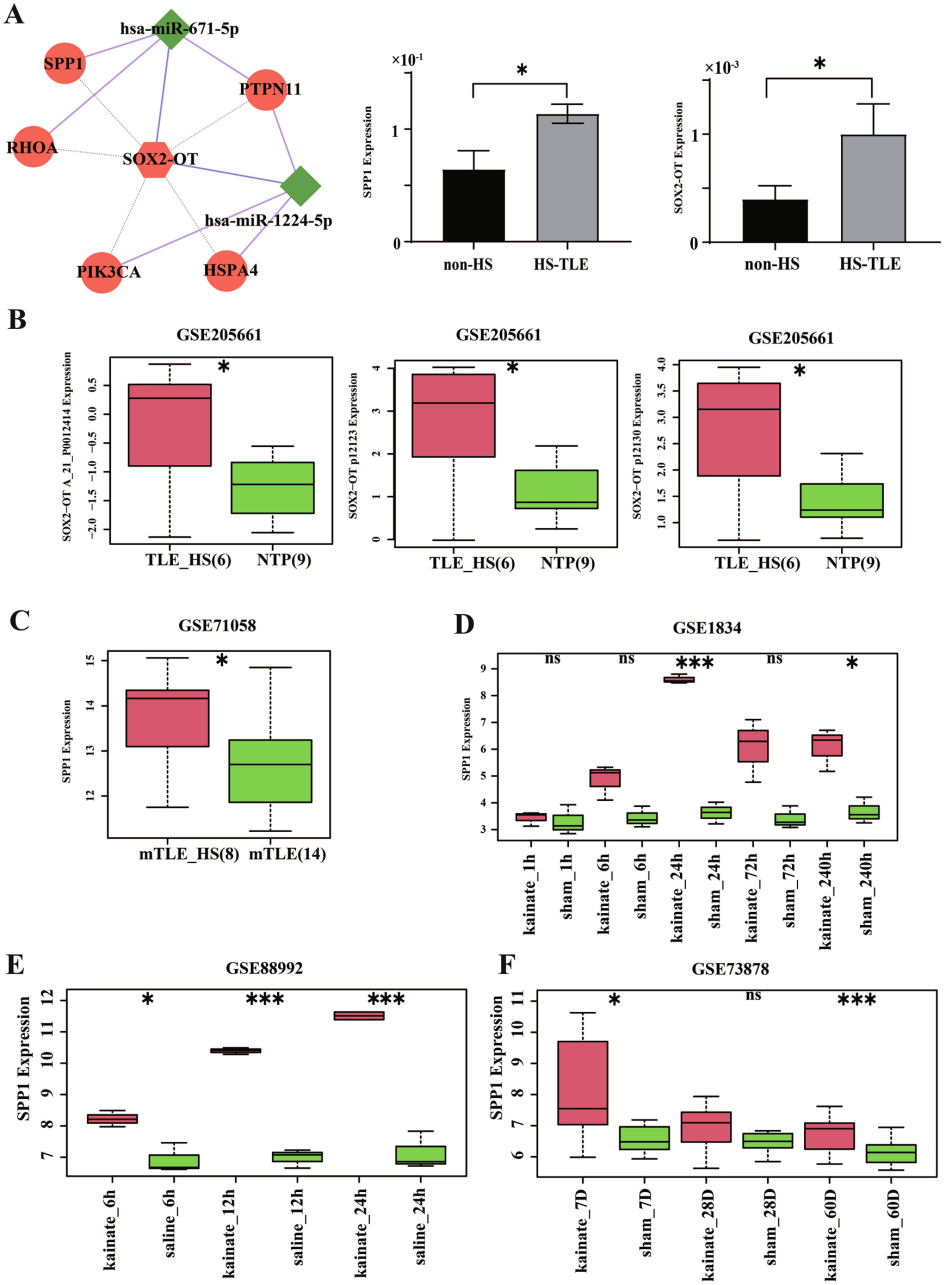
The validation of RNAs in key lncRNA regulatory network in hippocampal sclerosis. (**A**) A key lncRNA regulatory mechanism from ceRNA network. Hexagon represent DElncRNAs, whereas rhombus represent DEmiRNAs, and circles represent DEmRNAs. Right are the expression levels of SPP1 and SOX2-OT between non-HS and TLE-HS. (**B–F**) SOX2-OT was upregulated in GSE205661 (**B**), SPP1 was upregulated in GSE71058 (**C**), GSE1834 (**D**), GSE88992 (**E**) and GSE73878 (**F**).

As shown in Fig. 4A, the results of RT-PCR in our patients confirmed that SPP1 and SOX2-OT were significantly upregulated in the 3 TLE-HS patients compared with 3 TLE-nonHS patients in accordance with RNA-sequencing analyses.

To further verify the SOX2-OT/miR-671-5p/SPP1 axis in TLE-HS patients, we screened RNA sequencing data that compare TLE-HS with TLE-nonHS which collected from NCBI Gene Expression Omnibus (GEO) database, and analyzed the differential expression genes including SOX2-OT and SPP1. SPP1 was significantly upregulated in GSE71058 (5 patients with TLE-HS and 7 TLE patients without HS, Fig. 4B), while SOX2-OT was significantly upregulated in GSE205661 (6 patients with TLE-HS and 9 normal temporal or parietal cortices patients undergoing internal decompression for traumatic brain injury, Fig. 4C).

Then we used animal models to explore the impact of SOX2-OT/miR-671-5p/SPP1 in TLE-HS, which can provide a better understanding of the epileptogenesis. Among different chemicals used to induce an epileptic seizure in animals, kainic acid is the most commonly used agent to induce TLE-HS. In the rat models, SPP1 was significantly upregulated in GSE1834 ("2-hit" seizure model at 24 and 240 h after kainate-induced seizures at P30 compared to P15, Fig. 4D). Furthermore in the mice models, SPP1 was significantly upregulated in GSE88992 (intrahippocampal microinjection of kainate or saline-injected animals were decapitated 6, 12 or 24 h post injection, Fig. 4E) and GSE73878 (kainate or saline injected unilaterally into the dorsal hippocampus of 12 week old C57BL/6 J mice, and the ipsilateral and contralateral hippocampi were isolated 7 and 60 d later, Fig. 4F).

## Discussion

TLE-HS is a severe form of epilepsy and often presenting with refractory seizures^21^. However, we lack a complete understanding of the epileptogenic process. Recently, inflammation and immune responses have been increasingly implicated as potential underlying mechanisms in patients with TLE-HS^22–24^. Gene expression studies also suggest that immune pathway activation is a driving force in disease progression^3,25,26^. Based on the present study through bioinformatic analysis, we identified 15 important immune related genes indcluding HSP90AA1, SOD1, HRAS, JAK2, HSPA4, FOS, RHOA, PIK3CA, PTPN11, HSP90AB1, CDC42, PDGFB, RAC1, SPP1, IFNGR1 in TLE-HS patients, which may enrich the field for treating TLE-HS patients. Furthermore, based on enrichment analysis for targeted genes, we revealed five immune related pathways including “Th17 cell differentiation”, “Natural killer cell mediated cytotoxicity”, “B cell receptor signaling pathway”, “Chemokine signaling pathway”, “T cell receptor signaling pathway” in TLE-HS human samples. Th17 cells produce multiple inflammatory cytokines including IL-17, IL-21, IL-22, GM-CSF, and transcription factor retinoid-related orphan nuclear receptor γt^27^. NK cells possess natural cytotoxicity, antibody-dependent cellular cytotoxicity and also produce a plethora of cytokines, which take part in the regulation of the immune responses and can contribute to the pathogenesis of many immune mediated diseases^28^. B cell receptor is one of the most important receptors for B cells to sense their environment. In viral and bacterial infections, recognition of antigens derived from these pathogens induces proliferation and functional differentiation of B cells to effector subsets in periphery, which is essential process for host defense^29^. Chemokine activities are mediated through G-protein coupled receptors, which mediate transmission of stimuli as diverse as hormones, peptides, glycopeptides, and chemokines^30^. T cell signaling is important for efficient T cell development, activation, and immune tolerance, so TCR signaling dysregulation can thus lead to anergy or autoimmunity^31^. These five pathways could better understand immune mechanisms in TLE-HS, and pave the way for precise and effective immunotherapeutics.

It is predicted that perhaps 80% of the human genome is transcribed as non-coding RNA genes, which encoded in the genome but not translated into proteins. A total of 1726 DEmRNAs, 160 DElncRNAs, and 38 DEmiRNAs were identified across the transcriptomes in TLE-HS patients compared with TLE-nonHS group. In this paper, we identified SPP1, an extracellular secreted glycol phosphoprotein, significantly upregulated in the TLE-HS samples. Many studies have reported that aberrant expression of SPP1 was closely related to the tumor biology, such as proliferation, migration and invasion^32–34^. Taking a step further, we also found that SOX2-OT, short for SRY box transcription factor 2 overlapping transcript, was markedly up-regulated in TLE-HS through bioinformatics analysis. Accumulated evidence reports the crucial roles of SOX2-OT in the regulation of tumors, central nervous system development and ischemic heart failure^35,36^. In the ceRNA network theory, lncRNA can regulate mRNA by competitively sponging miRNA^37^. In our study, SOX2-OT connected to miR-671-5p, which upregulate the target genes SPP1 in TLE-HS patients. On the basis of public databases, we employed the transcriptomics analyses and key gene validation for TLE-HS patients vs control (GSE205661 and GSE71058), TLE-HS rat model vs control (GSE1834), and TLE-HS mouse model vs control (GSE88992 and GSE73878). Then we identified both SOX2-OT and SPP1 were significantly upregulated in these different databases including patients and animal models. Taken together, our results revealed that SOX2-OT/SPP1 can be explored for the immune related molecular signaling involving the onset, progression and prognostic in TLE-HS patients and animal models.

It is now widely accepted that seizures can occur in AD patients^38^ and AD related pathological changes might be a causative factor for late-onset unprovoked seizures^39^. As clinical data shown, the prolonged febrile seizures in children appear to damage hippocampus and later in life lead to HS^40^. In addition, the majority of epileptic encephalopathies develop secondary cognitive impairment due to the epileptic activity or the underlying etiology. In our study, a PPI cluster is related to neurodegenerative disease including “Pathways of neurodegeneration”, “Parkinson disease”, “Alzheimer disease”, “Neurotrophin signaling pathway”, which exemplify the probability that the epilepsy is related to neurodegenerative diseases.

Undeniably, our study has several limitations. Firstly, the sample size of each group in this study and the number of validated genes were rather small. So, multicenter cohort studies with larger sample sizes are needed in the future. Secondly, the sequencing technique may not attribute RNA changes to specific cell populations in the hippocampus. Therefore, the whole genome single-cell sequencing is needed in the future. Despite the limitations, our study constructed the first immune associated ceRNA network in human TLE-HS, which displayed regulatory pathway in TLE-HS patients for further investigation.

In summary, we used RNA-seq analysis to illustrate the landscape of molecules and pathways on human TLE-HS. Also, we found several differential immune related genes like HSP90AA1, SOD1, HRAS, JAK2, HSPA4, FOS, RHOA, PIK3CA, PTPN11, HSP90AB1, CDC42, PDGFB, RAC1, SPP1, IFNGR1. Further ceRNA regulatory network analysis found SOX2-OT connected to miR-671-5p and up-regulate the target gene SPP1. Taken together, the data provided a novel insight into the complex immune network of differential expressed RNAs in TLE-HS. The immune related SOX2-OT/miR-671-5p/SPP1 axis may be the potential therapeutic target for TLE-HS. However, further studies in serum or CSF samples in a larger cohort of patients are needed to validate the molecule mechanism.

## Conclusion

We found several differential immune related genes in TLE-HS, and we first suggest that immune related SOX2-OT/miR-671-5p/SPP1 axis could be a novel target for TLE-HS in both patients and animal models. However further in vivo experiments are necessary to better comprehend the mechanistic effects. These findings suggest that immune related pathway SOX2-OT/miR-671-5p/SPP1 axis should be explored as a potential therapeutic strategy for TLE-HS patients and animal models.

## Data Availability

All datasets generated for this study are included in the article. The figures in our paper are original for this article, and we have permission to use it.

## Abbreviations

TLE: Temporal lobe epilepsy
HS: Hippocampal sclerosis
ceRNA: Competitive endogenous RNA
GO: Gene ontology
KEGG: Kyoto encyclopedia of genes and genomes
DE: Differential expression
DEGs: Differential expression genes
IRGs: Immune related genes
RT-PCR: Real-time reverse transcription polymerase chain reaction
GEO: Gene expression omnibus
